# Challenges and Solutions in the Development of Genomic Biomarker Panels: A Systematic Phased Approach

**DOI:** 10.2174/138920212800793339

**Published:** 2012-06

**Authors:** K Shahzad, A Fatima, M Cadeiras, N Wisniewski, G Bondar, R Cheng, E Reed, MC Deng

**Affiliations:** 1 Department of Internal Medicine, Brody School of Medicine at East Carolina University, Greenville, NC 27834, USA; 2 Advanced Heart Failure Program, Division of Cardiology, Department of Medicine, David Geffen School of Medicine at UCLA, Los Angeles, CA 90095, USA; 3 UCLA Immunogenetics Center, Department of Pathology, David Geffen School of Medicine at UCLA, Los Angeles, CA 90095, USA

**Keywords:** Post-genome era, genomics, gene expression profiling, biomarker panels, molecular classifier, heart transplantation, multiorgan dysfunction, personalized medicine.

## Abstract

In the post-genome era, high throughput gene expression profiling has been successfully used to develop genomic biomarker panels (GBP) that can be integrated into clinical decision making. The development of GBPs in the context of personalized medicine is a scientifically challenging and resource-intense process. It needs to be accomplished in a systematic phased approach to address biological variation related to a clinical phenotype (e.g. disease etiology, gender, etc.) and minimize technical variation (noise). Here we present the methodological aspects of GBP development based on the experience of the Cardiac Allograft Rejection Gene Expression Observation (CARGO) study, a study that lead to the development of a molecular classifier for rejection screening in heart transplant patients.

## INTRODUCTION 

The completion of the Human Genome Project has revolutionized the field of biomedical sciences by providing an answer to the question of the genetic sequence of the human species [1]. Based on this development, availability of high throughput whole genome gene expression profiling (GEP) technology has opened new horizons to understand the complex biology of systems [2]. Microarrays provide the ability to study the expression of thousands of known and unknown genes in samples from patients at times of specific clinical events. DNA microarrays [3-6] have been used to study gene expression patterns retrospectively in biopsies from normal and rejecting pediatric renal allografts [7] and have defined differential patterns of gene activation associated with rejection as well as nephrotoxicity. Gene expression profiling of peripheral blood mononuclear cells (PBMC), where circulating cells capture the deranged physiology of organ systems, has been successfully used to characterize the systemic inflammatory response following intravenous endotoxin administration in healthy individuals [8]. It has also been used to characterize multiorgan dysfunction (MOD) following trauma [9], to diagnose sepsis [10], and to characterize the response to mechanical circulatory support device surgery, with inflammatory activation and T-cell suppression in stable post-operative patients [11]. 

Whole genome GEP technology has been successfully implemented to develop genomic classifiers to detect/predict outcomes in clinical medicine e.g. breast cancer [12,13], cardiac allograft rejection [14], and coronary artery disease [15,16]. In this article we will discuss the methodological aspects of genomic biomarker panel (GBP) development based on the experience of the Cardiac Allograft Rejection Gene Expression Observation (CARGO) study in the context of advancing post-genome era technology. We will use the exemplary phenotypes of heart transplant rejection (from our CARGO experience) and MOD (a challenging phenotype in our field of advanced heart failure) to elaborate the process of GBP development and validation. We will highlight the various challenges in the process and will suggest some possible solutions. 

## SYSTEMS-BIOLOGICAL CONCEPTS IN MEDICINE

The strategic plan in gene expression biomarker panel development can best be conceptualized in the framework of systems biology. *Systems biology* is a comprehensive quantitative analysis utilized to understand in which way all the components of a biological unit interact functionally over time. Systems approaches have long been taken, particularly in immunology, physiology, development, and neurobiology. However, technology development during the 1980s permitted the concepts generated by many years of reductionist inquiry to be analyzed in the context of the entire system. Automated DNA sequencers enabled the sequencing of genomes and the definition of polymorphisms among individuals; microarray analysis permitted global transcriptional profiling [17], and advances in mass spectrometry led to large-scale proteomic and metabolomic analysis. The plethora of data generated by these high-throughput platforms led to the rapid growth of computational biology and bioinformatics. Thus, knowledge of the complete sequences of genomes, together with technology that permit the monitoring of information flow leading to specific cellular functions, set the stage for systems biology [18-39]. The NIH-roadmap explicitly incorporates this approach as a necessary future research strategy [40]. 

**Fig. (1):**
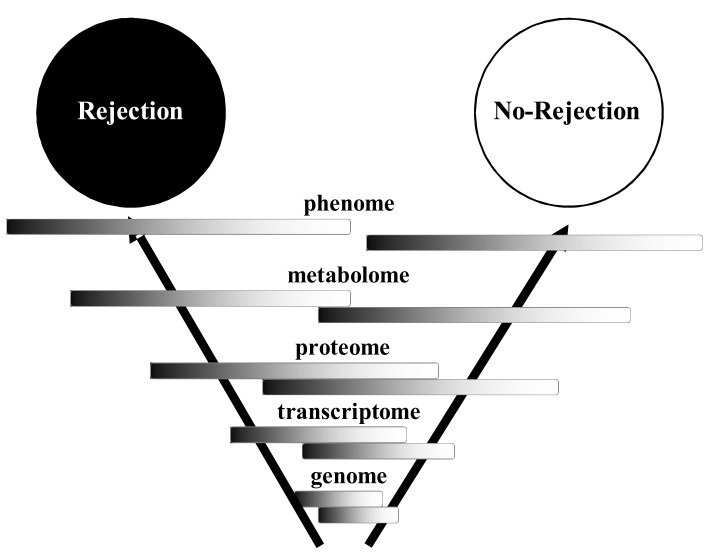
Systems biological perspective of relationship between genome, transcriptome, proteome, metabolome, and phenome.

Systems biological approaches include level distinction (also termed multi-scaling), component interaction, emergent properties, downward causation, and dynamic “feedback loop” behavior. *Emergent properties* are properties which are present on higher levels of a system, e.g. heart transplant rejection or multi organ dysfunction [MOD], but cannot be monocausally explained by the properties of the individual components on a lower system level, (e.g. individual gene transcripts). *Downward causation* are effects of properties on higher levels of the system, e.g. rejection of the heart or MOD on the properties on a lower system level, (e.g. individual gene transcripts). Dynamic “*feedback loop” behavior* is the probabilistic effect of iterative interactions of systems components, e.g. a set of gene transcripts, on the properties of other systems components, e.g. MOD. In essence, the systems biological approach postulates that *quantifiable differential expression patterns* of individual genes, proteins, and modules on the molecular and cellular level are simultaneously related, in a mathematically describable probabilistic way, to the clinical entity of interest on the highest systems level, the phenotype level (Fig. **1**). Within the *concept of multiscaling of different hierarchical levels*, the relationship between properties on the lower system level and emergent properties on the higher system level are key to understanding the phenotype within the concept of systems biology.

## CONCEPTUAL CHALLENGES

The major challenge in clinical research and genomic biomarker panel development is to control for circumstances unrelated to the relationship between transcriptome and phenome level [41]. Standardized operational procedures are required to control for and minimize epidemiological, technical, biological/physiological, and statistical variation, which is unrelated to the outcome. This principle is applied to every step of experimental design, conduct, data analysis and interpretation. Another challenge to the validity of the findings relates to the number of samples to be included in a study. In 2005 Michiels *et al.* published a re-analysis of seven publically available microarray studies which all had the goal of cancer outcome prediction [42,43]. The authors showed that the list of genes identified as predictors of prognosis was highly unstable, and molecular signatures strongly depended on the sample numbers in the training sets. It highlights the primary challenge in using microarray data, where one is patient-limited and gene-rich: whether genes and signatures are truly significant or whether they are products of random variation (i.e. over-fitted to noise) [44]. The newer more cost effective microarray platforms have greatly helped to overcome the problems of over-fitting. For appropriate power calculation in gene expression studies the described methods need to be followed [38,45].

## PHASED APPROACH

**Fig. (2):**
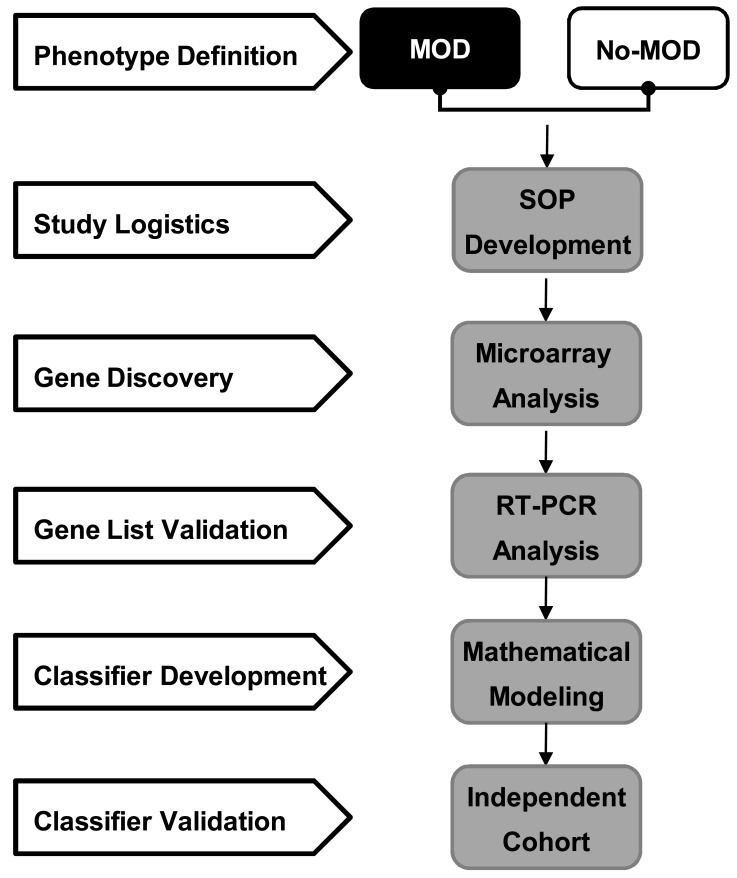
Approach to develop genomic diagnostic and prognostic classifiers/scores to detect/predict clinical outcomes divided into 1) phenotype definition, 2) establishment of study logistics, 3) gene discovery by microarray analysis, 4) differential gene list validation using RT-PCR, and 5) molecular classifier development using mathematical algorithm, and 6) external classifier validation in an independent patient population. **Footnotes:** MOD = presence of multiorgan dysfunction; No-MOD = absence of multiorgan dysfunction; SOP = standardized operating procedures; RT-PCR = real-time polymerase chain reaction.

Based on a clinically important phenotype of interest, GEP based biomarker panel development is conducted in well defined phases (Fig. **2**): (1) *Clinical phenotype consensus definition*; (2) *Establishment of study logistics*; (3) *Candidate gene discovery* using a combination of genome-wide and knowledge-base approaches; (4) *Differential gene list validation *using PCR assays; (5) *Molecular classifier algorithm development* using rigorous statistical methods; and (6) *External classifier validation* in an independent patient population. 

### Phase 1: Clinical Phenotype Consensus Definition

In medicine any clinical problem may elicit genomic test development. The consensus definition of the clinical phenotype of interest is critically important. Expectations toward a genomic test may include 1) identification of patients in the early stages of the development of the phenotype of interest, 2) differential diagnosis of the phenotype of interest from related phenotypes, 3) identification of patients at high risk for future development of a phenotype of interest, or 4) assessment of response to a specific therapy.

#### Imperfect Clinical Phenotype Standards 

Diagnostic test development requires a clinical standard against which to measure the new test’s performance. The existence of imperfect clinical standards creates a formidable challenge. For the correlation of clinical results with gene expression patterns it is crucial that the clinical phenotype is clearly defined. In the CARGO study the clinical standard was a biopsy-based histopathological assessment [14]. Biopsy sampling, however, is subject to sampling error, as technical procedures may vary from center to center and also from clinician to clinician. The ensuing histopathological assessment by a trained pathologist is subject to inter-observer-variability [46]. The same was observed in the CARGO-study with concordance between core pathologists of 60% for moderate/sever rejection [47]. To minimize the impact of inter-observer and inter-center variability in determining the clinical phenotype, an approach with the panel of independent (“central”) investigators blinded to clinical information needs to be adopted for the appropriate selection of samples for gene expression studies. In analogy, to develop reliable phenotype comparators for the challenging phenotype of MOD, currently available best practice MOD-scoring systems [48-53] have to be utilized.

#### Dichotomous vs. Continuous Phenotype Choices 

Dichotomous phenotypes, typically with extreme ends of a clinical spectrum, with fully developed signs/symptoms on one end and a contrasting picture with no clinical signs/symptoms at the other end, are more likely to be associated with differential gene expression signatures and therefore are more appropriate choices for a proof-of-principle study. Although for this proof-of principle rationale a design based on a dichotomous phenotype is preferable, the choice of a continuous phenotype spectrum may allow for a more practical outcome validation approach. In the phenotype of MOD where continuous scoring systems are used to grade the degree of organ dysfunction e.g. Sequential Organ Failure Assessment (SOFA) score [52,53], extreme scores can be used for the development of GBP to identify the right candidate genes associated with the disease. This GBP can be applied to assess the intermediate levels of organ dysfunction later on during validation.

#### Static vs. Dynamic Phenotypes 

Clinical disease phenotypes have historically been described as static entities by the presence or absence of characteristic clinical signs/symptoms and molecular markers of a disease process. While in fact the state of health and disease in human body is a dynamic phenomenon, static phenotype choices are relatively less challenging to assess the associated gene expression patterns. Characterizing the dynamics of a disease process in an organism is intrinsically more difficult than quantifications rooted in static features. One needs to assess and monitor the changes in a biological system over time using time series analysis of GEP. 

### Phase 2: Establishment of Study Logistics

After the consensus definition of the – dichotomous or continuous – phenotype of interest, the establishment of multicenter study logistics is important especially in critical care medicine. 

#### Multicenter Study Design 

A multicenter study design is recommended for genomic studies, it increases sample size but also variabilities. For example, in the CARGO-study, eight US-centers were involved. 

#### Standardized Operating Procedures 

**Fig. (3):**
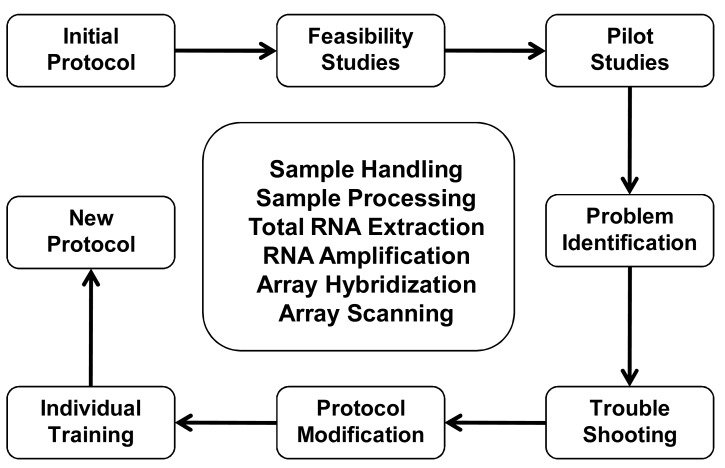
Approach to develop consensus standardized operating procedures (SOP) for genomic studies including 1) development of initial protocol, 2) feasibility studies, 3) pilot studies, 4) problems identification, 5) troubleshooting, 6) protocol modification, 7) individual training, and 8) implementation of the new protocol.

In general, sample processing and array analysis should be organized by consensus standardized operating procedures (SOP). Any SOP should be tested for validity in pilot studies (Fig. **3**). Pilot or feasibility studies can be helpful in the early identification of problems in the collection, handling and processing of specimens before a larger study is undertaken. These pilot studies may also help in determining the new processes and personnel training needs required before the implementation of a new protocol. For example, the CARGO-study team paid clear attention that the clinical trial protocol addressed microarray-related aspects of sampling, sample processing and storage. The SOP was distributed to the participating centers and it required agreement upon by everyone involved, including clinicians and bioinformaticians. It specified in detail how biopsies should be taken and how blood should be drawn, including the tube type, storage temperature and storage duration. Only centers which agreed on these specifications were allowed to participate in the study. This process must take place well before the first sample is taken, and also include that each center is informed on the technologies that are applied to their samples [54]. 

For genomic studies, specimen collection protocols have special requirements for preservation of macromolecules and/or analytics of interest. In addition to specimen type, things to consider when planning to collect specimens include: the collection method, the collection tubes or containers needed, the population that will provide the specimen, personnel required to collect the specimen (and training in the collection process), the distance from the collection point to the processing lab and to the storage facility (if this is a different location), stabilizing or preservation techniques for maintaining/preservation of macromolecules required for the specific analyses, and specimen labeling and tracking strategies. 

#### Whole Blood vs. PBMC Approach 

Recently the whole blood approach was suggested which reduces the handling steps involved in sample processing but leads to contamination with Globin-mRNA from the RBCs which decreases transcript detection sensitivity and increases signal variation. Methods have been reported to reduce this contamination up to 70% [55] and gene expression platforms e.g. Affymetrix®, are available for improved array results using this approach. The comparative performance of these protocols still needs to be validated. If comparable results can be achieved, whole blood approach will be the best approach because of its advantage over the cell separation approach to minimize the technical noise. 

#### Core Microarray Facility 

For microarray experiments samples need to be processed at a core microarray facility, which follows a specific SOP for each sample. Processing of all samples in batches, in a single microarray laboratory, ideally by one technician, limits the technical noise induced by handling of the samples by several individuals or by using different workstations in different laboratories. On the first level of clinical biomarker panel development one would like to avoid too much unwanted systematic technical noise. 

### Phase 3: Candidate Gene Discovery

After establishing the multicenter infrastructure with motivated centers and established logistics for the sample processing the actual experimental phase of gene discovery is critical.

#### Microarray Based Non-Supervised Approach 

A variety of strategic decisions in the gene discovery process need to be made. The *whole-genome* wide approach provides more comprehensive scanning to identify significant and novel genes which were not described before in the clinical context of interest. Using different bioinformatics tools e.g. significance analysis of microarray (SAM), GeneSpringGX, and Partek, differentially expressed genes are identified in relationship to the *concurrent phenotype* of interest.

#### Knowledge Based Approach 

The knowledge-base approach, focusing on known genes, may ignore new biology which might be apparent in a non-hypothesis-driven approach. Although the knowledge-based approach leads to the identification of genes that can distinguish the different phenotypes of interest, whole genome arrays may yield additional, different, or better gene candidates.

Both approaches can be combined with a leukocyte microarray and a knowledge-base approach or literature review as adopted in previous studies [13,14]. The goal is to find a set of genes that could be reproducibly measured by RT-PCR in an RNA preparation. 

#### Effect of Genetic Mutations 

**Fig. (4):**
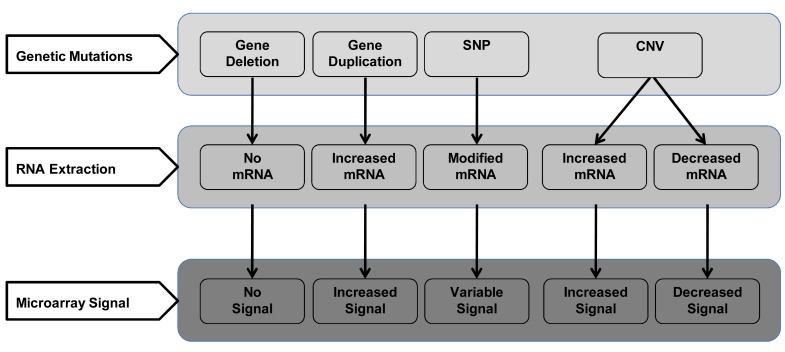
Effect of some basic types of genetic mutations on gene discovery in GBP development. **Footnotes:** SNP = single nucleotide polymorphism; CNV = copy number variation.

The presence of various genetic variations have effect on transcriptome [56]. The process and mechanisms of how these variations effect the gene expression is not well understood. One possible way to assess the effect of these variations i.e. gene deletion, gene duplication, single nucleotide polymorphism (SNP), and copy number variations (CNV) could be to assess their effect on the mRNA extracted and its detection by available techniques during gene discovery (Fig. **4**). Through these effects on the transcriptome, these genetic variations can affect the results of not only gene discovery but also their validation and later clinical application of GBP. With the lack of current better understanding of the relationship between genetic variations and the transcriptome, it is difficult to address this challenge.

It has to be noted that in the GBP strategy proposed here, the emphasis is on assembly of expression profiling based candidate genes while purposefully neglecting the additional variation associated with various genetic variations on the genome level as well as the modulatory effect on the proteome level.

### Phase 4: Differential Gene List Validation/Verification

#### Realtime-PCR Validations 

Since the array-based non-supervised approach to discover differentially expressed genes is limited by sensitivity and variability, a second molecular biological method such as RT-PCR with higher sensitivity and lower variability needs to be applied to internally validate the discovery phase gene list. For realtime-PCR validation studies, genes which do not discriminate between absence and presence of the phenotype of interest are being considered as normalization genes. Additional assays should be included as controls to detect genomic DNA contamination by the difference between a transcribed and non-transcribed region of a gene e.g. the Gus-B gene and a spiked-in control template to determine if the PCR reaction was successful, e.g. the Arabidopsis gene [14]. 

The differences observed between the microarray and PCR methods in significance of specific genes may be due to 1) lower sensitivity of microarrays leading to the elimination of genes which show discrimination in PCR; 2) enhanced reproducibility of RT-PCR allowing measurement of small differences in gene expression [57], likely undetectable by microarrays (usually eliminating genes that show <1.5 - to 2-fold changes). While a complete list validation by RT-PCR is the most complete approach, it is also the most resource intense approach. Based on the level of significance (false discovery rate (FDR) <0.05) and the available resources the gene list can be reduced to a small list for the external validation/testing studies.

#### NanoString nCounter Gene Expression System 

Recently digital mRNA quantification technology (NanoString nCounter analysis system) has been suggested to be a potential tool which does not require the enzymatic reactions as compared to real time PCR. This exciting new technology which currently is not using a whole transcriptome approach may be utilized for the validation step during classifier development instead of the RT-PCR approach after the initial gene discovery by array technology [58]. 

#### Next Generation Sequencing

The next-generation sequencing (NGS), with direct sequencing and quantification of DNA and RNA, is opening new avenues for discovery in the post-genome era [59]. The technology is available as RNA-Seq for expression profiling. Currently, it has been shown to have comparable results with microarrays [60] and can be used for the gene discovery. Further developments in the technology may allow it as a future replacement for the RT-PCR as a new validation tool for the GBPs. 

### Phase 5: Molecular Classifier Algorithm Development

The goal for the development of a diagnostic classifier is the systematic reduction of the internally validated differentially expressed gene list into a minimum number algorithm which then is subjected to external validation/training in Phase 6. 

#### Discriminatory vs. Classifier Genes 

One must differentiate between the identification of discriminatory genes and the development of a robust classifier. For example in the CARGO-study, several cytotoxic T-cell genes were not selected for the classifier by the automated and unbiased statistical method of linear discriminant analysis, while PDCD1, a known marker of T cell activation, whose expression is correlated with the other T-cell genes was chosen in the final model. The fact that some of the “well-known” markers of this pathway were not selected by the method points to the strong relevance of PDCD1 as the optimal representative of this pathway and suggests that these other genes were either not incrementally informative or lacked sufficient reproducibility to be included in the model. 

#### Independent Testing of Selected Classifier Genes 

To test the performance of the selected classifier genes, collected patient samples are typically randomly split into separate *training and validation sets*. The significantly differentially expressed genes identified in the training set and are tested in a validation set to estimate the degree of misclassification. Alternatively, the *leave-one-out cross validation* approach can be used which involves using a single observation from the original sample as the validation data, and the remaining observations as the training data. This is repeated such that each observation in the sample is used once as the validation data. 

#### Mathematical Modeling 

The classifier score development poses unique mathematical modeling challenges. Conceptually, correlation strategies [61] and mutual information-strategies [62,63] need to be distinguished. The methods for analyzing gene expression data included principal components analysis, linear discriminant analysis (LDA, StatSoft Inc.), logistic regression (SAS Institute Inc.), Prediction Analysis of Microarrays (PAM), voting, classification and regression trees (TreeNet, Salford Systems), Random Forests, nearest shrunken centroids and k-nearest neighbors. LDA constructs a linear classifier by automatically selecting genes and/or metagenes [64] that, in combination, optimally separate rejection and quiescent samples in the training set. The robustness of selected genes and the appropriate number of genes in the classifier were both evaluated by cross-validation. 

#### Biological Plausibility of the Classifier/Test Gene List 

An important challenge is in understanding how genomic results correlate to known or “expected” biological pathways for the disease state. Biological plausibility is a valid criterion to increase one’s confidence in a genomic result, but the converse—the lack of such information—is *not* evidence that a genomic result is false since the biological literature is not complete in this regard. In fact, genomic technologies enable researchers to discover new genes or pathways associated with disease that may not be expected from the existing literature. If only “expected” genes are accepted in genomics studies, new discovery will be stunted [14]. 

#### Selection of Score Cutoff 

After constructing the diagnostic test score with specific range using rigorous mathematical modeling (e.g. 0-40 for CARGO classifier) Receiver Operator Characteristic (ROC) curve analysis is performed to define the best possible cutoff on this range with desired sensitivity, specificity, negative predictive value (NPV), positive predictive value (PPV) in relationship to the phenotype of interest using Area Under the Curve (AUC) as a criteria to estimate how well the classifier performs.

### Phase 6: External Classifier Validation/Testing

The external clinical validation of the constructed diagnostic classifier in an independent patient sample is critically important in genomic test development. 

#### Independence of Primary Clinical Validation Cohort 

After the initial training set of samples during the test development, it is mandatory to validate these results in an independent patient sample for the generalizability of the classifier performance. For example, in the CARGO-study, an independent cohort of CARGO patients was selected to validate the effectiveness of the LDA classifier defined in the Diagnostic Development phase using a prospective and blinded study protocol. The primary objective of the validation study was to test the prespecified hypothesis that the diagnostic score distinguishes absence of rejection, defined as ISHLT grade 0 from moderate/severe biopsy-proven acute rejection, defined as ISHLT grade ≥3A, both grades determined from local and centralized cardio-pathological examination. This was assessed using a two-tailed Student’s t-test for comparing score distributions for rejection and quiescent samples. Secondary and exploratory objectives included documentation of diagnostic performance across thresholds and description of correlations to clinical variables.**

#### Clinical Test Replicability 

In order to establish the test reproducibility and to demonstrate acceptable precision of the Test results further studies need to be designed to study the effect of different variables associated with the specimen handling process which may cause variation in test results e.g. operator-to-operator variation, run-to-run variation, lot-to-lot variation of reagents, plate-to-plate variation, and section-to-section variation of the plates used to run the test.**

## COMPARISON AGAINST STANDARD OF CARE & PERSONALIZED USE 

While in the first step after GBP development, test performance should be based on cross-sectional phenotype identification by the GEP-test [65], in a second step required for full clinical acceptance [66], the GBP can be tested against the standard of clinical care e.g. endomyocardial biopsy (EMB) for heart transplant rejection monitoring. For heart transplant rejection monitoring, this comparison was performed between invasive EMB approach and the non-invasive GBP approach in the Invasive Monitoring Attenuation through Gene Expression (IMAGE) trial. The results showed that the GBP monitoring approach lead to a significant reduction in the number of invasive biopsies and increased patient satisfaction in a low risk heart transplant patient population [67,68]. 

In the last step, the added information derived from intra-individual temporal/longitudinal profiling increase the values of rule out, detection or prediction goals [69] and allows to enter into the personalized approach as compared to population based clinical scoring systems. In a case report we have described the existence of such a relationship between the heart transplant GBP and the organ level functions [70].

## CONCLUSIONS

The scientific advancements in the post human genome project era have revolutionized the field of biomedical sciences. Using the available GEP technology has been successfully used to develop biomarker panels for different phenotypes in clinical medicine. The process of GBP development is associated with various conceptual, clinical, methodological, and computational challenges. These challenges in the GBP development can be best addressed by adopting the described novel systematic phased approach.
